# Epidemiology, Presentation, Management and Outcomes in Chronic Inflammatory Demyelinating Polyneuropathy in Birmingham, UK: The Impact of Ethnicity

**DOI:** 10.1111/jns.70065

**Published:** 2025-09-29

**Authors:** Zeinab Rajabally, Lydia Spencer, Niraj Mistry, Yusuf A. Rajabally

**Affiliations:** ^1^ Department of Neurology University Hospitals Birmingham Birmingham UK; ^2^ Aston Medical School Aston University Birmingham UK

**Keywords:** Asian, Bangladeshi, chronic inflammatory demyelinating polyneuropathy, epidemiology, ethnicity, Indian, Pakistani

## Abstract

**Background:**

Whether ethnicity impacts on epidemiology, presentation, management, and outcome is unknown in chronic inflammatory demyelinating polyneuropathy (CIDP).

**Methods:**

We studied the prevalence/incidence of CIDP in Asian (Indian/Pakistani/Bangladeshi) and white subjects in Birmingham, UK, and associations of ethnicity with demographics/deprivation/phenotype/treatment and outcomes.

**Results:**

On 10th July 2025, CIDP prevalence was 6.18 per 100 000 (95% CI: 4.66–8.05). Prevalence was lower in Asian (Indian/Pakistani/Bangladeshi) compared to white subjects (2.64 per 100 000 vs. 10.15 per 100 000; RR: 0.260, 95% CI: 0.111–0.609; *p* < 0.001). Prevalence in ≥ 50‐year‐olds was lower in Asian (Indian/Pakistani/Bangladeshi) compared to white subjects (8.00 per 100 000 vs. 46.68 per 100 000; RR: 0.172; 95% CI: 0.061–0.479; *p* < 0.001) but similar in 18–49‐year‐olds (2.48 per 100 000 vs. 1.83 per 100 000; RR: 1.355, 95% CI: 0.273–6.712; *p* = 0.661). Mean incidence of CIDP was 0.54 per 100 000 per year (95% CI: 0.404–0.713). CIDP incidence was lower in Asian (Indian/Pakistani/Bangladeshi) than in white subjects (0.24 per 100 000 per year vs. 0.86 per 100 000 per year, RR: 0.278; 95% CI: 0.118–0.654; *p* = 0.002). Asian (Indian/Pakistani/Bangladeshi) ethnicity was independently associated with younger age (*p* = 0.037), greater social deprivation (*p* = 0.045), and noncompliance to treatment (*p* = 0.016). No association of Asian (Indian/Pakistani/Bangladeshi) ethnicity was found with CIDP sub‐type, diagnostic delay, pretreatment disability, access to high‐cost therapies, or posttreatment outcomes.

**Conclusions:**

Subjects of Asian (Indian/Pakistani/Bangladeshi) ethnicity in the UK may have a lower risk of CIDP after 50 years of age, but an equivalent risk between 18 and 49 years, compared to white subjects. They may present younger, be more socially deprived, and be more likely noncompliant to treatment, compared to white subjects.

## 1 Introduction

Inter‐ethnic differences have been reported as accounting for disparities in presentation, treatment, and outcome of several neurological disorders [1, 2, 3, 4]. In peripheral neuropathy, inter‐ethnic differences have mainly been studied in the setting of diabetic polyneuropathy, with a demonstrated lower risk for some ethnic groups [5, 6, 7].

Chronic inflammatory demyelinating polyneuropathy (CIDP) is the commonest autoimmune neuropathy. Considering other autoimmune neurological diseases, lower incidence rates have been described in the UK in nonwhite subjects compared to white subjects for multiple sclerosis (MS), whereas no difference was observed for myasthenia gravis [8]. Earlier onset and greater disease severity have otherwise been reported for MS in Asian migrants to the US [9] and in North African migrants to France [10]. A recent large UK study, however, found no association between ethnic background and MS severity [11]. Interethnic differences in presentation and outcome have also been reported in Neuromyelitis Optica Spectrum Disorder [12]. In relation to the impact on disease management, ethnic disparities have been reported in myasthenia gravis care [13, 14] as well as in MS care in a recent UK study [15].

To our knowledge, inter‐ethnic differences have not been studied in CIDP. The low prevalence of CIDP represents a challenge for ascertaining eventual differences in the characteristics of affected subjects from ethnic minority subgroups. Our center in Birmingham, UK, offers the possibility of attempting to study this question, representing the only subspecialist unit for inflammatory neuropathy in the wider region and serving a city with a large nonwhite population, having reached 51.4% of the total population in 2021, of which the majority (27.1%) are of Asian (Indian, Pakistani, or Bangladeshi) origin. In the current study, we mainly aimed to (i) ascertain the prevalence and incidence rates of CIDP in subjects of Asian (Indian/Pakistani/Bangladeshi) ethnicity, in comparison to white subjects, (ii) determine associations of Asian (Indian/Pakistani/Bangladeshi) ethnicity with demographic features, social deprivation, clinical characteristics, treatment access, and outcomes.

## 2 Materials and Methods

We retrospectively reviewed electronic records of consecutive adult subjects (age ≥ 18 years) with a diagnosis of CIDP or possible CIDP, meeting European Academy of Neurology/Peripheral Nerve Society (EAN/PNS) 2021 criteria, attending the Inflammatory Neuropathy Service, University Hospitals Birmingham, UK. The Inflammatory Neuropathy Service is the only referral center for adults with chronic inflammatory neuropathy for the city of Birmingham and receives referrals for all newly diagnosed cases locally. It also receives additional out‐of‐area tertiary referrals from the West Midlands region and beyond. We collected demographics, ethnicity data, place of residence, social deprivation category, disease sub‐type, mode of onset, pretreatment disease duration from symptom onset, treatment(s) administered, treatment compliance, and outcomes.

Ethnicity data were separated into two main sub‐categories: (i) any nonwhite ethnicity, (ii) any white ethnicity. We specifically compared, in the current study, from the former sub‐category, Asian (specifically, Indian/Pakistani/Bangladeshi) ethnicity and any white ethnicity in the prevalence and incidence analyses. Population data and estimated ethnicity proportions per age group were obtained from the 2021 UK Census, Office of National Statistics [16] and through the 2021 Census Profile for Birmingham [17]. Pretreatment disability was evaluated through the Overall Neuropathy Limitation Score (ONLS) [18]. The ONLS scale is systematically used at all clinic attendances in our practice (5 points for upper limb score, 7 points for lower limb score; optimal score of 0). Posttreatment outcome was defined as that best achieved with treatment and determined through the posttreatment ONLS, the posttreatment inflammatory Rasch‐built Overall Disability Scale (I‐RODS) [19], and through the “CIDP Treatment‐Response Category” (“CT‐RC”) [20]. The CT‐RC is graded 1–5: (i) CT‐RC 1: “complete response” (corresponding to full recovery), (ii) CT‐RC 2: “good partial response” (equivalent to at least having the ability to do all common self‐care tasks and the ability to walk without aid), (iii) CT‐RC 3: “moderate partial response” (equivalent to at least having the ability to do most but not all common self‐care tasks and the ability to walk with unilateral support), (iv) CT‐RC 4: “poor partial response” (equivalent to at least having purposeful upper limb movements without the ability to perform any common self‐care task and the ability to walk with bilateral support), (v) CT‐RC 5: “unresponsive” (corresponding to no or no meaningful change from pretreatment level). Social deprivation was assessed by ascertaining for each subject, through house number, street name, and postcode, the English Index of Multiple Deprivation 2019 (IMD 2019), which provides a set of relative measures of deprivation for small geographical areas based on 7 domains: (i) income deprivation, (ii) employment deprivation, (iii) education, skills, training deprivation, (iv) health deprivation and disability, (v) crime, (vi) barriers to housing and services, (vii) living environment deprivation [21]. The lowest IMD decile (decile 1) corresponds to the 10% most deprived areas and the highest (decile 10) to the 10% least deprived. The postcode of residence was, in addition, categorized as local (Birmingham) or nonlocal (non‐Birmingham). Traveling distance was ascertained from place of residence to the hospital. Noncompliance with treatment was defined as any of the following: (i) declining offered treatment for CIDP, (ii) requesting alternative treatment or treatment dosage alteration for CIDP against medical advice, and (iii) undergoing any treatment for CIDP against medical advice.

Statistical analyses were performed with SPSS 28.0 (Armonk, USA). CIDP point prevalence rate was determined at the point of analysis for the Birmingham residents and separately for Asian (Indian/Pakistani/Bangladeshi) and for white subjects, and stratified per age group (18–49 years and ≥ 50 years). Mean yearly incidence over the study period was determined for the Birmingham cohort and in subjects of Asian (Indian/Pakistani/Bangladeshi) and of white ethnicity. Relative risk (RR) for CIDP was established by nonparametric one‐sample binomial tests and 95% confidence intervals (CI) through Clopper‐Pearson (exact) tests for Asian (Indian/Pakistani/Bangladeshi) ethnicity versus white ethnicity. The prevalence, incidence, and RR analyses included only Birmingham residents and used available local population ethnicity data and age group proportions [16]. This was performed as over 50% of patients seen at our tertiary center reside outside the area in different regions with variable population proportions for different ethnic sub‐groups, which render their inclusion in epidemiological analyses impossible. Associations were studied through Spearman's Rank correlation, and independent relationships were established through linear regression. In view of the exploratory nature of this analysis, correction for multiple correlations was not performed. Significance was set at *p* < 0.05 for all tests.

This analysis was conducted as part of a registered and approved retrospective clinical audit of the impact of social and economic factors on the diagnosis and management of CIDP in subjects attending the Inflammatory Neuropathy Service, University Hospitals Birmingham, UK (CARMS‐22724, 16th January 2025). Audit does not require Ethics Committee approval in the UK.

## 3 Results

**FIGURE 1:**
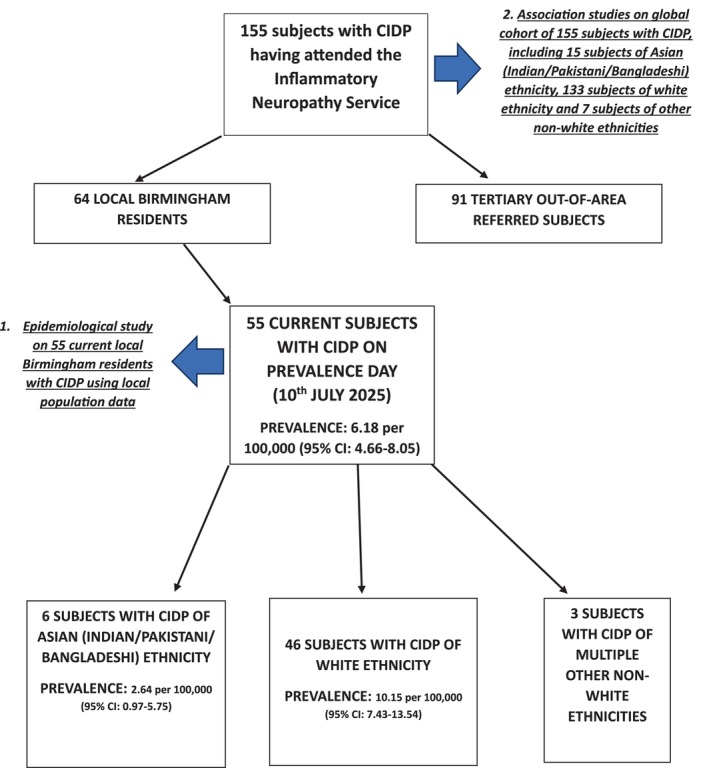
Identification and breakdown of cases of CIDP having attended the Inflammatory Neuropathy Service, University Hospitals Birmingham between July 2014 and July 2025.

Figure 1 shows the breakdown of the total cohort studied and details the analyses performed: (1) epidemiological study and (2) whole population association studies. The total cohort consisted of 155 consecutive subjects (58 females, 97 males) meeting EAN/PNS criteria for CIDP or possible CIDP, having attended our service between July 2014 and July 2025. Twenty‐two (14.2%) subjects were of nonwhite ethnicity, of whom 15/22 (68.2%) were “Asian (Indian/Pakistani/Bangladeshi)” and 7/22 (31.8%) were of 4 other different nonwhite ethnicities.

Epidemiological results for the sub‐cohort of Birmingham residents (55 subjects; 6 Asian (Indian/Pakistani/Bangladeshi), 46 white, 3 of other nonwhite ethnicities) are detailed in Table 1. On 10th July 2025, the point prevalence of CIDP in Birmingham was 6.18 per 100 000 (95% CI: 4.66–8.05). Point prevalence was lower in Asian (Indian/Pakistani/Bangladeshi) subjects compared to white subjects (2.64 per 100 000 vs. 10.15 per 100 000; RR: 0.260, 95% CI: 0.111–0.609; *p* < 0.001). Point prevalence in subjects aged 18–49 years was lower than that in subjects aged ≥ 50 years (2.20 per 100 000 vs. 33.93 per 100 000, RR: 0.065; 95% CI: 0.032–0.133; *p* < 0.001). Point prevalence in subjects aged ≥ 50 years was lower in subjects of Asian (Indian/Pakistani/Bangladeshi) ethnicity compared to subjects of white ethnicity (8.00 per 100 000 vs. 46.68 per 100 000; RR: 0.172; 95% CI: 0.061–0.479; *p* < 0.001). However, prevalence rates in subjects aged 18–49 years were similar in Asian (Indian/Pakistani/Bangladeshi) and white subjects (2.48 per 100 000 vs. 1.83 per 100 000; RR: 1.355, 95% CI: 0.273–6.712; *p* = 0.661). The mean yearly incidence of CIDP over the 11‐year study period in Birmingham was 0.54 per 100 000 per year (95% CI: 0.404–0.713). Incidence in Asian (Indian/Pakistani/Bangladeshi) subjects was lower than in white subjects (0.24 per 100 000 per year vs. 0.86 per 100 000 per year, RR: 0.278; 95% CI: 0.118–0.654; *p* = 0.002).

**TABLE 1:** Epidemiology of CIDP in Birmingham, UK; point prevalence on 10th July 2025 and mean yearly incidence over an 11‐year period (2014–2025): Stratification by ethnicity and age.

	Total population of Birmingham (all ethnicities): 1 144 923		RR for Asian (Indian/Pakistani/Bangladeshi) vs. White (95% CI)	*p*
	Asian (Indian/Pakistani/Bangladeshi)	White (Any white ethnicity)	NA	
Total population	309 853	556 608	NA	
18–49 years population	162 983	218 190	NA	
≥ 50 years population	64 449	233 775	NA	
Total global prevalence (all ethnicities)	6.18 per 100 000 (95% CI: 4.66–8.05)		NA	
Prevalence by ethnicity	2.64 per 100 000 (95% CI: 0.97–5.75)	10.15 per 100 000 (95% CI: 7.43–13.54)	RR: 0.260 (95% CI: 0.111–0.609)	*p* < 0.001
Total global prevalence 18–49 years (all ethnicities)	2.20 per 100 000 (95% CI: 1.01–4.18)		NA	
Prevalence in 18–49 years by ethnicity	2.48 per 100 000 (95% CI: 3.00–8.96)	1.83 per 100 000 (95% CI: 0.67–3.98)	RR: 1.355 (95% CI: 0.273–6.712)	*p* = 0.661
Total global prevalence ≥ 50 years (all ethnicities)	33.93 per 100 000 (95% CI: 24.84–45.26)		NA	
Prevalence in ≥ 50 years by ethnicity	8.00 per 100 000 (95% CI: 2.18–20.5)	46.67 per 100 000 (95% CI: 33.35–63.55)	RR: 0.172 (95% CI: 0.061–0.479)	*p* < 0.001
Global incidence	0.54 per 100 000 per year (95% CI: 0.404–0.713)		NA	
Incidence by ethnicity	0.24 per 100 000 per year (95% CI: 0.09–0.52)	0.86 per 100 000 per year (95% CI: 0.64–1.16)	RR: 0.278 (95% CI: 0.118–0.654)	*p* = 0.002

The main characteristics of Asian (Indian/Pakistani/Bangladeshi) subjects with CIDP in the whole cohort of 155 subjects are summarized in Table 2. Fifteen of 155 subjects (9.68%) were of Asian (Indian/Pakistani/Bangladeshi) ethnicity. There were 5 females and 10 males (ratio of 1:2). The mean age of Asian (Indian/Pakistani/Bangladeshi) subjects was 52.6 years (S.D.: 15.4), and the mean disease duration pretreatment initiation was 23.4 months (SD: 40.9). Ten Asian (Indian/Pakistani/Bangladeshi) subjects (66.7%) had typical CIDP, and 5 (33.3%) had variant CIDP. Of those with variant forms, 3 (20%) had multifocal CIDP (Lewis Sumner syndrome), and 2 (13.3%) had motor CIDP. Two Asian (Indian/Pakistani/Bangladeshi) subjects (13.3%) had an associated co‐morbidity impacting on physical function, and 2 (13.3%) had concurrent diabetes.

**TABLE 2:** Main characteristics of 15 subjects of Asian (Indian/Pakistani/Bangladeshi) ethnicity with CIDP having attended the Inflammatory Neuropathy Clinic, University Hospitals Birmingham, UK between July 2014 and July 2025.

Mean age‐years (SD)	52.6 (15.4)
Gender F:M (ratio)	5:10 (1:2)
Mean pretreatment disease duration‐months (SD)	23.4 (40.9)
CIDP disease sub‐type	– Typical: 10/15 (66.7%) – Variant focal/multifocal: 3/15 (20%) – Motor: 2/15 (13.3%)

Results of correlation studies within the total cohort are summarized in Table 3. Asian (Indian/Pakistani/Bangladeshi) ethnicity correlated with younger age (*p* = 0.015) and greater social deprivation (*p* = 0.009). Linear regression analysis showed that Asian (Indian/Pakistani/Bangladeshi) ethnicity was independently associated with both younger age (*p* = 0.037) and social deprivation (*p* = 0.045). Asian (Indian/Pakistani/Bangladeshi) ethnicity did not correlate with gender (*p* = 0.733), postcode location (*p* = 0.322), traveling distance to the hospital (*p* = 0.169), disease subtype proportions (*p* = 0.298), acute‐onset presentations (*p* = 0.128), pretreatment disease duration (*p* = 0.173), pretreatment ONLS (*p* = 0.230), treatment with immunoglobulins (*p* = 0.895), treatment with plasma exchange (*p* = 0.547), treatment with corticosteroids (*p* = 0.509), use of immunosuppressants (*p* = 0.157), posttreatment ONLS (*p* = 0.729), amplitude of ONLS improvement (*p* = 0.130), posttreatment I‐RODS (*p* = 0.883), CT‐RC (*p* = 0.912), co‐morbidities with functional impact *(p* = 0.192), associated diabetes (*p* = 0.619), or time to maximal improvement (*p* = 0.125). Noncompliance to treatment correlated with Asian (Indian/Pakistani/Bangladeshi) ethnicity (*p* < 0.001), having a local Birmingham postcode (*p* = 0.021), and posttreatment I‐RODS (*p* = 0.02). An independent association of noncompliance to treatment was found on linear regression with all three co‐variates: Asian (Indian/Pakistani/Bangladeshi) (*p* = 0.016), local Birmingham postcode (*p* = 0.048), and posttreatment I‐RODS (*p* = 0.016).

**TABLE 3:** Spearman's Rank correlations and independent associations (through linear regression) of Asian (Indian/Pakistani/Bangladeshi) ethnicity with relevant co‐variables, in 155 consecutive subjects with CIDP attending University Hospitals Birmingham, UK.

	Spearman's rho	*p*	Independent association with Asian (Indian/Pakistani/Bangladeshi) ethnicity
Age	−0.194	*p* = 0.015	YES (*p* = 0.037)
Gender	−0.028	*p* = 0.733	NA
Disease sub‐type proportions	−0.084	*p* = 0.298	NA
Acute onset presentation	0.123	*p* = 0.128	NA
Postcode Location	0.080	*p* = 0.332	NA
Traveling distance to the hospital	−0.111	*p* = 0.169	NA
IMD2019 decile	−0.208	*p* = 0.009	YES (*p* = 0.045)
Pretreatment disease duration	−0.110	*p* = 0.173	NA
Pretreatment ONLS	0.097	*p* = 0.23	NA
Posttreatment ONLS	−0.028	*p* = 0.729	NA
ONLS improvement	0.12	*p* = 0.13	NA
Posttreatment I‐RODS	−0.015	*p* = 0.883	NA
CT‐RC	0.009	*p* = 0.912	NA
Co‐morbidities with functional impact	−0.105	*p* = 0.192	NA
Associated diabetes	−0.040	*p* = 0.619	NA
Use of IVIg	−0.011	*p* = 0.895	NA
Use of PLEX	0.049	*p* = 0.547	NA
Use of corticosteroids	0.053	*p* = 0.509	NA
Use of IS	0.114	*p* = 0.157	NA
Time to maximal improvement	0.127	*p* = 0.125	NA
Noncompliance to treatment	0.320	*p* < 0.001	YES (*p* = 0.016)

Abbreviations: CT‐RC: CIDP Treatment‐Response Category; IMD 2019: Index of Multiple Deprivation 2019 decile; I‐RODS: Inflammatory Rasch‐Built Overall Disability Scale; IS: immunosuppression; IVIg: intravenous immunoglobulins; NA: nonapplicable; ONLS: Overall Neuropathy Limitation Score; PLEX: plasma exchange.

## 4 Discussion

Inter‐ethnic differences have been demonstrated in several neurological disorders. There is, however, a paucity of data in CIDP, likely in view of its rarity. There have been a limited number of epidemiological studies performed in CIDP outside Europe and North America [22, 23, 24], and comparative inter‐ethnic analyses have, to our knowledge, not been performed in heterogeneous cohorts, nor in India, Pakistan, or Bangladesh, where most of the nonwhite subjects in our cohort were originally from.

Through a large cohort of subjects with CIDP attending our tertiary center and serving the second largest city in the UK, which now has a majority nonwhite population of high Asian (Indian/Pakistani/Bangladeshi) proportion, we have attempted to study the impact of ethnicity in CIDP. Although it is possible that few patients with CIDP may not have been referred to our service, we believe the prevalence rate found is likely accurate in the absence of other subspecialist services for inflammatory neuropathy locally and in the wider region and the well‐established links and referral pathways with local neurologists, which result in the systematic referral of all newly diagnosed local cases. The point prevalence and incidence rates are, furthermore, in keeping with those previously found in the UK [25] and similar to those recently reported in the Netherlands [26]. We found that the prevalence and incidence of CIDP were significantly lower in Asian (Indian/Pakistani/Bangladeshi) subjects compared to white subjects. Although the analysis of the Asian (Indian/Pakistani/Bangladeshi) sub‐group was from a limited total population of around 300 000, this was comparable in population size to that done in Iceland recently [27]. We found similar prevalence rates in Asian (Indian/Pakistani/Bangladeshi) subjects and white subjects aged 18–49 years, contrasting with a significantly lower prevalence rate in Asian (Indian/Pakistani/Bangladeshi) subjects aged ≥ 50 years compared to white subjects. The explanation for this age‐dependent variable CIDP risk in Asian (Indian/Pakistani/Bangladeshi) subjects is uncertain and requires further study. In MS, disease risk has long been described as increased by migration in childhood from low‐ to high‐incidence areas [28]. MS risk has been reported in Canada as increasing in migrant populations with the proportion of life spent in the country, suggesting an acculturation effect [29] and the role of environmental exposures [30]. Geographical and ethnic epidemiological variations are, on the other hand, not known in CIDP. Our observations in this cohort may suggest that exposure to possible modifiable risk factors such as diet, obesity, smoking, or infections, as described in MS [31], may diminish with age in Asian (Indian/Pakistani/Bangladeshi) subjects, as opposed to white subjects, thereby reducing disease risk later in life. Inter‐ethnic dietary differences may, specifically, represent an important avenue for further investigation. A study from Italy showed a reduced risk of CIDP in subjects consuming rice at least three times weekly and fish at least once weekly [32]. Such dietary habits may interestingly be more common in Asian (Indian/Pakistani/Bangladeshi) subjects than in white subjects [33] and may have been protective. It is also finally possible that protective genetic factors may have a greater role later in life against CIDP, the prevalence of which increases with age.

Previous studies have reported a possible protective effect of Asian ethnicity against diabetic polyneuropathy compared to white subjects [6, 34] and subjects of Arab ethnicity [35]. Differences in adiposity and its distribution, as well as height, have been postulated as potential explanations [36], although other mechanisms, including inflammatory processes, may be in play. Of relevance, a higher risk of diabetes is reported in subjects with CIDP [37], but no association of ethnicity with concurrent diabetes was found in our cohort, possibly in part due to the younger age of Asian (Indian/Pakistani/Bangladeshi) subjects. Whether, and through which eventual mechanisms, Asian (Indian/Pakistani/Bangladeshi) ethnicity may be protective against CIDP requires further study.

We found correlations of Asian (Indian/Pakistani/Bangladeshi) ethnicity with younger age and with greater social deprivation in the wider cohort which included out‐of‐area tertiary referred subjects. Independent associations were found with both factors. These findings are in keeping with existing population data which show lower mean age as well as greater deprivation in nonwhite, and Asian populations, compared to white populations in the UK [38]. No significant differences were noted in disease sub‐type proportions or acute‐onset presentations. Of note, sensory forms of CIDP were not observed in Asian (Indian/Pakistani/Bangladeshi) subjects, while affecting 7/133 (5.3%) of subjects of white ethnicity. We found no differences in pretreatment disease duration and pretreatment disability in Asian (Indian/Pakistani/Bangladeshi) subjects compared to white subjects of the cohort. The frequency of use of all main therapeutic agents, including immunoglobulins, corticosteroids, plasma exchange and immunosuppressants, was otherwise similar in Asian (Indian/Pakistani/Bangladeshi) and white subjects. The amplitude of functional improvement as well as posttreatment outcomes, as determined through three measures, were also similar in both ethnic groups. These findings suggest comparable disability level at presentation, similar diagnostic delay, as well as equivalent management modalities and outcomes, irrespective of ethnicity, in the whole cohort. This differs from what has been reported in the UK to date in other chronic diseases, where greater severity [3], longer delay to treatment [39], and poorer outcomes [40, 41], have been described, in ethnic minority populations. In view of the well‐documented effects of structural barriers to care offered to ethnic minorities [42], it is possible that the rarity of CIDP and the centralized care within a single sub‐specialist center in the city and region, may have contributed to equity of care in this cohort.

Noncompliance to treatment was associated with Asian (Indian/Pakistani/Bangladeshi) ethnicity and having a local Birmingham postcode, with independent associations for both. Poor treatment compliance has been reported in ethnic minority and inner‐city populations in various medical settings in the UK [43, 44, 45, 46, 47]. Multiple factors, including cultural, linguistic, and religious, may be involved and impact upon communication with medical teams, understanding of choice, benefits, and risks of therapeutic options on offer, as well as potentially directly affecting individual decision‐making processes. All these factors may ultimately result in reduced access to specialist care for ethnic minorities as well as subsequently impact on patient/physician interactions within secondary and tertiary settings. Ethnicity‐related differences in illness perception have also been documented. For instance, African American and Hispanic patients in the US have been shown to report fewer and less severe symptoms in diabetic polyneuropathy compared to white patients [48]. If patients perceive their symptoms as less severe, they may view aggressive treatment as unnecessary, highlighting the importance of thorough and culturally sensitive communication about the treatable nature of CIDP. Implicit bias, whereby healthcare professionals are influenced by unconscious racial prejudice, has otherwise been previously reported to impact patient and clinician communication and therefore affect clinical decision‐making [49]. Language barriers, even when interpreters are used, can reduce the quality of clinician–patient interaction. Previous reports have suggested that in interpreted consultations, clinicians tend to spend less time listening and make fewer supportive statements, increasing the risk of miscommunication [50]. In conclusion, our findings highlight the increasing value of “cultural competence” in clinical practice in ethnically diverse settings [51], as well as the need for early and adequate training of clinical staff in communication skills to improve healthcare delivery to ethnically diverse populations [52]. Noncompliance to treatment was, of note, independently associated with poorer posttreatment I‐RODS, highlighting the importance of efforts in improving this aspect of care to improve outcomes.

Our study is limited by its single‐center and retrospective design. We were not able to investigate epidemiological characteristics of other individual ethnic minorities, in view of the rarity of CIDP and small numbers of affected subjects from several other minority ethnic sub‐groups. Furthermore, the prevalence and incidence analyses were performed versus subjects of “any white ethnicity,” which represents itself a heterogeneous group. We cannot exclude, in view of the small sample size, that the Asian (Indian/Pakistani/Bangladeshi) subjects with CIDP in our cohort may not be representative of their wider ethnic group, with regard to detailed deprivation level, educational background, and disease awareness. This may have impacted upon our findings particularly with regard to predisease disability or tertiary referral rate, as we have recently described in another recent study [53]. Our analysis did also unfortunately not include more detailed analysis of co‐morbidities, nor the potential impact of place of birth or time/age of arrival in the UK, or of linguistic difficulties in Asian (Indian/Pakistani/Bangladeshi) subjects, all of which may be of relevance [54, 55, 56]. In addition, comparative inter‐ethnic data on patient impression of change and health‐related quality of life measures were not available.

Despite limitations, we believe our study brings novel insights into the epidemiology of CIDP in different ethnic sub‐groups in the UK, which merit further investigation in larger diverse cohorts. Future studies are needed to confirm if, similar to diabetic polyneuropathy, CIDP risk is lower in Asian (Indian/Pakistani/Bangladeshi) subjects compared to white subjects after 50 years of age and to understand why the risk may be equivalent in‐between ethnic groups earlier in life. Finally, and more generally, our findings on noncompliance to treatment and its deleterious effects on outcomes highlight the need for careful consideration of cultural factors in the holistic management of chronic neuromuscular diseases like CIDP in diverse ethnic environments.

## Conflicts of Interest

Y.A.R. has received consultancy honoraria from Sanofi, Argenx, Janssen, LFB, Polyneuron, Grifols, Takeda, Dianthus, Vitaccess, has received educational sponsorships from LFB and CSL Behring, and has obtained research grants from LFB. Z.R., L.S., and N.M. have no conflicts of interest.

## Data Availability

The data that support the findings of this study are available on request from the corresponding author. The data are not publicly available due to privacy or ethical restrictions.
